# Identification, antifungal resistance, and genomic characterization of a single *Candida auris* isolate from urinary tract infection

**DOI:** 10.3389/fcimb.2025.1641542

**Published:** 2025-09-10

**Authors:** Jie Li, Ziheng Wang, Rui Zheng, Yangyan Wang, Xin Guo, Xiaoning Li, Peng Zhang

**Affiliations:** Department of Laboratory Medicine, The First Affiliated Hospital of Wannan Medical College (Yijishan Hospital of Wannan Medical College), Wuhu, Anhui, China

**Keywords:** *Candida auris*, antifungal resistance mechanisms, genomic characterization, database, whole-genome sequencing

## Abstract

**Objective:**

To analyze the identification, antifungal resistance, and genomic characteristics of a *Candida auris* (*C. auris*) strain isolated from a urine specimen of an ICU patient at Yijishan Hospital, Wannan Medical College, Anhui Province.

**Methods:**

The isolate was identified by Matrix-Assisted Laser Desorption/Ionization Time-of-Flight Mass Spectrometry (MALDI-TOF MS). The susceptibility of the isolates to fungi was determined by measuring the Minimum Inhibitory Concentration (MIC) values using the VITEK 2 COMPACT system. Whole-genome sequencing (WGS) was performed using high-throughput technology. Resistance and virulence genes were annotated using public databases, including NCBI (https://www.ncbi.nlm.nih.gov/, version 2.2.28), DFVF (http://sysbio.unl.edu/DFVF/, version 1.0), PHI-base (http://www.phi-base.org, version 5.0) and KEGG (https://www.kegg.jp/, version 89.1). A phylogenetic tree was constructed through analysis of the 18S rRNA nucleotide sequence.

**Results:**

The isolate named CAS20503 was identified as *C. auris*. Antifungal susceptibility testing showed resistance to fluconazole and amphotericin B. Genomic analysis identified resistance genes including *ERG11* (azole), *FKS1* (echinocandin), *ERG3* (polyene), and efflux pumps *CDR1*, *MDR1*. Resistance mutations were detected. The virulence genes analyzed based on the DFVF database included *CaNik1*, *CHS2*, *DUR1,2*, *HSP90*, *ICL1*, *PMT1*, *PMT2*, *PMT4*, *SSD1*, *TPS2*. The host pathogenic genes identified by comparison with the PHI-base database included *CaCHS1, ADE2, FAS2, PMR1, CaTPS2, Tfp1.* KEGG annotation showed enrichment in infectious disease pathways. The phylogenetic tree constncted based on the nudeotide sequence analysis of 18S rRNA indicated that this strain (Genome accession number: JBPYFS000000000) exhibited a high degree of genomic similarity to the *C. auris* strain (Genome accession number: CP157510.1), which was isolated in ltaly in 2024 and belonged to clade l (a subset of the South Asian clade).

**Conclusion:**

Through an in-depth analysis of strain CAS20503, which was isolated from a urinary tract infection specimen at a tertiary public hospital in Anhui Province (Yijishan Hospital, Wannan Medical College), this study elucidated the drug resistance profiles and genomic characteristics of *C. auris*. The findings have provided critical evidence for the early identification, diagnosis, and optimization of antifungal therapeutic regimens for infections caused by this pathogen in clinical practice.

## 1 Introduction

*C. auris* is an emerging global microorganism. Through nosocomial transmission, it has spread to over 50 countries across six continents, rapidly garnering attention from global health authorities and emerging as a worldwide threat (Kim et al., 2024). The distinctive characteristics of *C. auris* have resulted in its inclusion in the “critical priority” category of the World Health Organization (WHO) Fungal Priority Pathogens List (Kriegl et al., 2025). This pathogen is characterized by high-level resistance to multiple antifungal agents and is associated with mortality rates ranging from 29% to 53% (World Health Organization, 2022). *C. auris* infection can cause severe invasive infections (Khatamzas et al., 2019). Global data indicate that among cases of fungal infections caused by *C. auris*, the axilla, groin, and nostril are the primary isolation sources, accounting for 29.7% of all isolated strains. The next most frequent sources are blood (17.0%), urine/urinary catheters (14.8%), and skin (13.6%). Notably, from 2022 to 2024, the proportion of isolates from urine/urinary catheters worldwide increased, making it the second most common isolation source in 2024 (19.3%) (La Bella et al., 2025). In recent years, *C. auris* has been recognized as a pathogen responsible for urinary tract infections (UTIs) on a global scale (Fazeli et al., 2019). The first public report on *C. auris* in urine dates back to 2010–2014, during which researchers collected urine isolates from a population in India (Kathuria et al., 2015). This study analyzed the drug resistance profiles and genetic characteristics of *C. auris* isolated from a urinary tract infection specimen of an elderly male patient, thereby providing insights into the diagnosis and treatment of fungal infections in the urinary system.

## 2 Information and methods

### 2.1 Patient information

The *C. auris* strain (CAS20503) utilized in the experiment was isolated from a urine sample of a patient with a urinary tract infection. A 62-year-old male patient presented with persistent right knee pain for over two months, accompanied by limb weakness for one week. He had a long-standing history of hypertension, diabetes mellitus, renal insufficiency, and hemodialysis. Following admission, the patient was transferred to the ICU due to respiratory failure. Five days later, given symptoms of fever and oliguria, a urine sample was collected for relevant examinations. Routine urinalysis revealed 3+ white blood cells. A midstream morning urine specimen was submitted for pathogen culture, which detected *C. auris*. Following a multidisciplinary consultation, the patient was administered intravenous caspofungin (70 mg as a loading dose, followed by 50 mg daily) in combination with flucytosine (100 mg/kg daily) based on fungal susceptibility testing results. One week after initiation of treatment, no leukocytes were detected in the urine. By the third week of treatment, urine fungal culture yielded negative results.

### 2.2 Instruments and reagents

Microbial identification and susceptibility testing were performed using the MALDI-TOF MS and VITEK 2 COMPACT systems (bioMérieux, France). Sabouraud dextrose agar medium and CHROMagar Candida chromogenic media were purchased from Jinan Baibo (China). Gram staining reagents were obtained from Zhuhai DL Biotech (China). Additional materials included target plates, formic acid, matrix solution, and fungal susceptibility panels (AST-YST) (bioMérieux, France). Genomic DNA was extracted using the Rapid Fungi Genomic DNA Isolation Kit (Shanghai Sangon Biotech, China). The Hieff NGS Ultima Pro DNA Library Prep Kit was used for library preparation (Yeasen Biotechnology Co., Ltd, China).

### 2.3 Culture and identification

Midstream urine was aseptically collected and immediately processed. Samples were inoculated onto Sabouraud dextrose agar medium and CHROMagar Candida chromogenic media and incubated at 35°C for 48 hours. A fungal smear was prepared from the colony, followed by Gram staining of the smear, followed by observation of fungal morphology under a microscope. For MALDI-TOF MS identification, colonies were applied to a target plate, treated with formic acid to lyse fungal cell walls, overlaid with matrix solution, and subjected to mass spectrometric analysis.

### 2.4 Antifungal susceptibility testing

Fungal colonies cultured for 48 hours were collected and adjusted to a 2.0 McFarland standard suspension. Disposable plastic tubes were placed in a rack, each containing 3 ml of 0.45% sodium chloride solution. Subsequently, 280 μl of the 2.0 McFarland suspension was added to each tube and mixed thoroughly. The AST-YST antifungal susceptibility cards were retrieved from the refrigerator, allowed to equilibrate to room temperature for 15–20 minutes, and then inserted into the inoculated tubes. The tubes were transferred to the VITEK 2 COMPACT system for automated determination of MIC.

To date, no specific susceptibility breakpoints have been established for *C. auris*. Therefore, this experiment utilized the tentative MIC breakpoints recommended by the U.S. Centers for Disease Control and Prevention (CDC) as reference standards (Centers for Disease Control and Prevention): fluconazole (FLC) ≥ 32 µg/mL; amphotericin B (AMB) ≥ 2 µg/mL; caspofungin (CSF) ≥ 2 µg/mL; micafungin (MFG) ≥ 4 µg/mL. For antifungal agents lacking CDC breakpoints (voriconazole (VRC) and flucytosine (5-FC), the breakpoints for the genus *Candida* specified in the Clinical and Laboratory Standards Institute (CLSI) M27-S3 protocol were adopted (voriconazole ≥ 4 µg/mL; flucytosine ≥ 32 µg/mL), which are applicable to *C. auris* (Ramos et al., 2025) (**Table 1**).

**Table 1:** List of types of drug susceptibility, reference standards and drug susceptibility results.

Antifungal agents	Breakpoints/Standard Source	MIC	Susceptibility Results
fluconazole	≥ 32 µg/mL/CDC	MIC ≥ 64μg/ml	R
amphotericin B	≥ 2 µg/mL/CDC	MIC = 8.0μg/ml	R
caspofungin	≥ 2 µg/mL/CDC	MIC ≤ 0.12μg/ml	S
micafungin	≥ 4 µg/mL/CDC	MIC ≤ 0.06μg/ml	S
voriconazole	≥ 4 µg/m/(CLSI) M27-S3	MIC = 0.25μg/ml	S
flucytosine	≥ 32 µg/mL/(CLSI) M27-S3	MIC ≤ 1μg/ml	S

“S” stands for susceptible; “R” stands for resistant.

### 2.5 Whole genome sequencing

The sequencing platform used was DNBSEQ-T7, with library preparation performed using the Hieff NGS Ultima Pro DNA Library Prep Kit; paired-end sequencing was employed, with an average read length of 150 bp, generating raw data containing 31,911,388 total reads (4,786,708,200 bp in total) and achieving 99.997% genome coverage. To ensure sequencing data quality, multiple quality control methods were implemented: Fastp software was used to analyze raw data quality, including base quality values (Q20 ratio 98.63%, Q30 ratio 95.87%) and GC content (45.15%); adapter-containing sequences were removed, with adapters accurately identified via read overlap regions; global trimming and sliding window quality trimming were performed (window length 4, with windows of quality below Q20 trimmed); base inconsistencies in overlap regions were corrected; low-quality sequences (reads containing more than 40% of bases with quality below Q15) and paired-end reads shorter than 35 nt were filtered out; and sequence trimming was conducted using Trimmomatic (version 0.36). Additionally, 10,000 reads were randomly selected and aligned with the NT database for species proportion counting, revealing that 99.52% of reads matched *C. auris* (indicating an extremely low contamination rate). Post-quality control, the data retention rate reached 99.94%, with the proportion of low-quality sequences at 0.0%, sequences containing excessive Ns at 0.057%, and short sequences at 0.003%, results that indicated high data purity and good integrity.

### 2.6 Bioinformatics analysis

#### 2.6.1 Drug resistance gene alignment

The target genes included *ERG11*, *FKS1*, *ERG3*, *CDR1*, and *MDR1*. Reference sequences and their accession numbers were retrieved from the NCBI database as follows: *ERG11* (Gene ID: 40026303), *FKS1* (Gene ID: 40026130), *ERG3* (Gene ID: 40028956), *CDR1* (Gene ID: 40025314), and *MDR1* (Gene ID: 40029187). Sequence alignment was performed using NCBI BLAST (version 2.2.28) and SnapGene (Version 8.0.3). For the alignment process, the whole-genome nucleic acid sequences of the test strains were aligned against the aforementioned NCBI reference sequences, and sequence matching patterns were analyzed, including completely matched genes, genes with insertion/deletion variations, and genes with point mutations.

#### 2.6.2 Virulence gene alignment

First, the gene prediction tool Prokka (version 1.10) was used to identify coding sequences (CDS) from the assembled genome, and the corresponding protein sequences were obtained. NCBI BLAST (version 2.2.28) was then employed to align these gene-encoded protein sequences against the DFVF V1.0 database. The screening criteria for virulence gene results were set as follows: E-value = 0 and query coverage > 60%.

#### 2.6.3 Pathogenic gene alignment

NCBI BLAST was used to align the gene-encoded protein sequences against the PHI-base V5.0 database. The screening criteria for pathogenic gene results were defined as E-value = 0 and query coverage > 95%.

#### 2.6.4 Database metabolic pathway classification

The protein sequences predicted in the above steps were converted to FASTA format (a total of 6530 CDS were predicted in this project). The KEGG Automatic Annotation Server (KAAS, version 2.1) was used as the core tool to associate genes with KEGG (version 89.1) data. Standardized protein sequences were batch-uploaded to the KAAS server (https://www.genome.jp/kaas-bin/kaas_main), with the corresponding species classification (fungi) and reference genome database (KEGG Orthology, ko) selected. Using BLASTP alignment, KAAS conducted homology searches between the input protein sequences and KEGG’s KO database to determine the KO number for each gene. The KO number serves as a key identifier linking genes to metabolic pathways. Output results included each gene’s KO number, corresponding metabolic pathway information (Pathway ID), and functional descriptions. The number of genes annotated in each metabolic pathway was counted, and a bar chart was generated.

### 2.7 Method for constructing the phylogenetic tree

Phylogenetic analysis was performed based on the 18S rRNA homologous sequence of the CAS20503 strain. This sequence was aligned against the NCBI BLAST database with the parameter set to identity > 90%, yielding a total of 19 included sequences. No outgroup was used for rooting the phylogenetic tree, as the strain had already been identified as *C. auris* by mass spectrometry. Using MEGA12 software (Version 12.0.9), a phylogenetic tree was constructed from these sequences via the maximum likelihood method with 1000 bootstrap replications.

### 2.8 Ethical approval

The fungal strain used in the experiment was isolated and cultured from a urine specimen of a clinical patient. Informed consent has been obtained from the subject for the use of the experimental specimen.

## 3 Experimental results

### 3.1 Morphological characteristics

**Figure 1:**
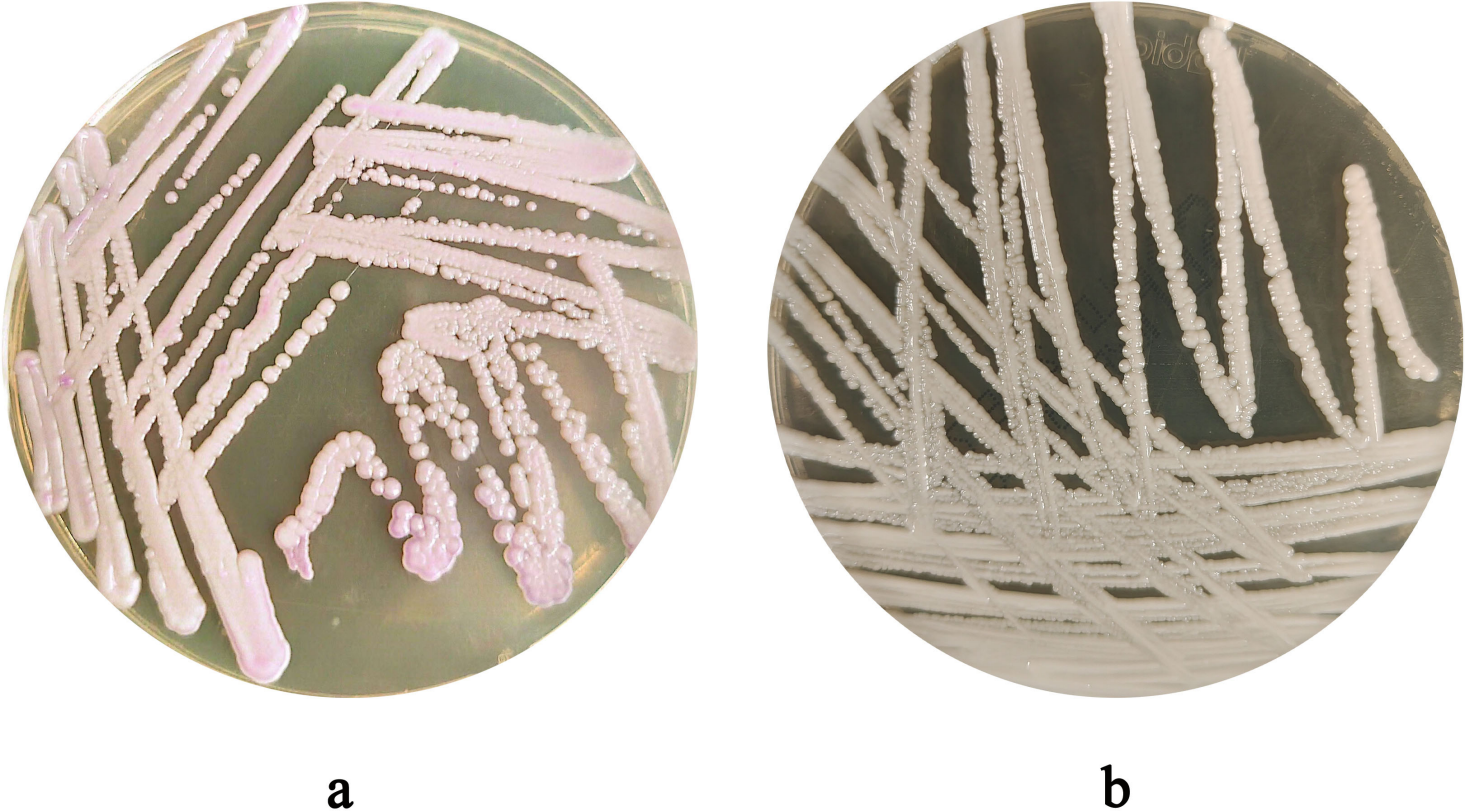
**(a)** Colony Morphology of *C. auris* strain CAS20503 Incubated on Sabouraud dextrose agar medium for 48 hours. **(b)** Colony Morphology of *C. auris* strain CAS20503 Incubated on CHROMagar Candida chromogenic media for 48 hours.

**Figure 2:**
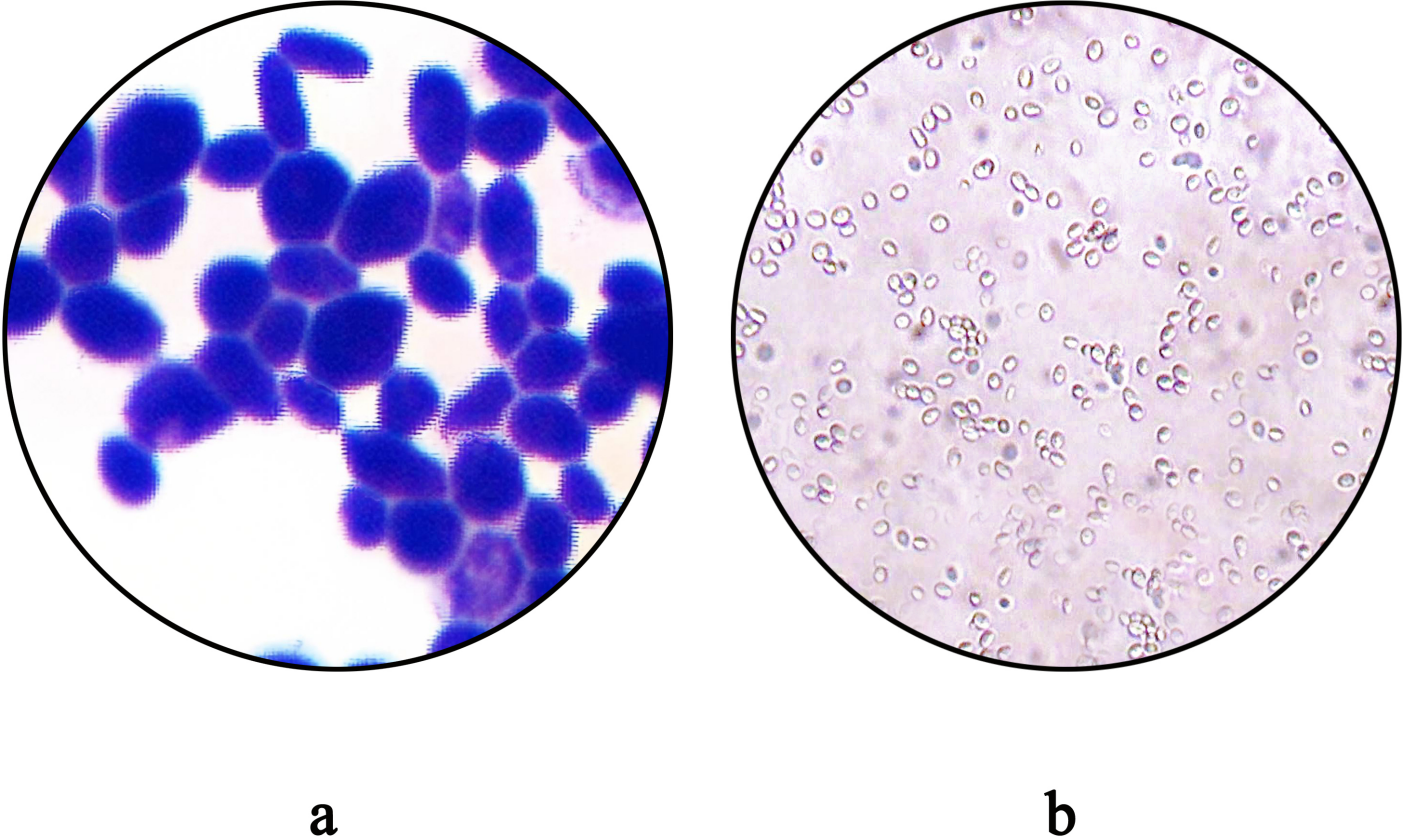
**(a)** Morphological Characteristics of *C. auris* strain CAS20503 Observed by Gram Staining Microscopy. **(b)** The microscopic characteristics of the wet mount of *C. auris* strain CAS20503 fungal colony suspension treated with 10% potassium hydroxide (KOH) were as follows.

After 48 hours of incubation, on CHROMagar Candida chromogenic medium, colonies were initially white, gradually transitioning to a pinkish-purple hue with prolonged incubation (**Figure 1a**). Colonies on Sabouraud dextrose agar medium appeared creamy white (**Figure 1b**). With Gram staining, under the microscope, the fungus could be observed as purple, oval yeast-like cells with a diameter of 3-6 μm (**Figure 2a**). Wet mount microscopic examination of the fungal suspension prepared with 10% potassium hydroxide (KOH) revealed the presence of budding spores in the fungus (**Figure 2b**).

### 3.2 Strain identification

**Figure 3:**
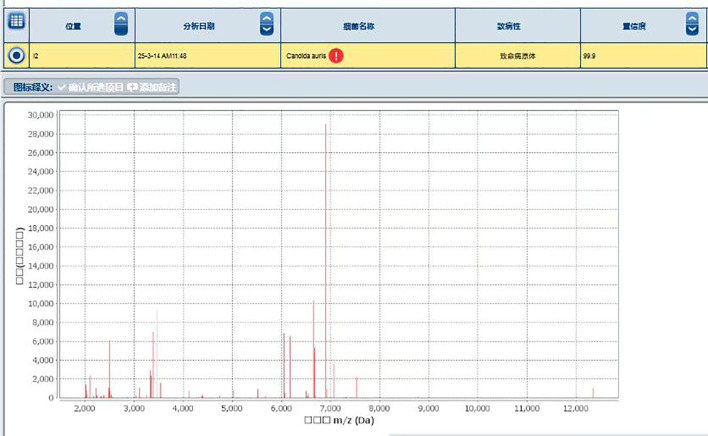
Identification of the Protein Fingerprint of *C. auris* strain CAS20503 Using MALDI-TOF MS. Peaks are visible across 2,000 to 12,000 m/z (Da) range, with significant peaks around 3,000 and 7,000 m/z, indicating protein masses.

MALDI-TOF MS analysis identified the isolate as *C. auris* with a high confidence score (99.9%) (**Figure 3**).

### 3.3 Antifungal susceptibility profile

**Figure 4:**
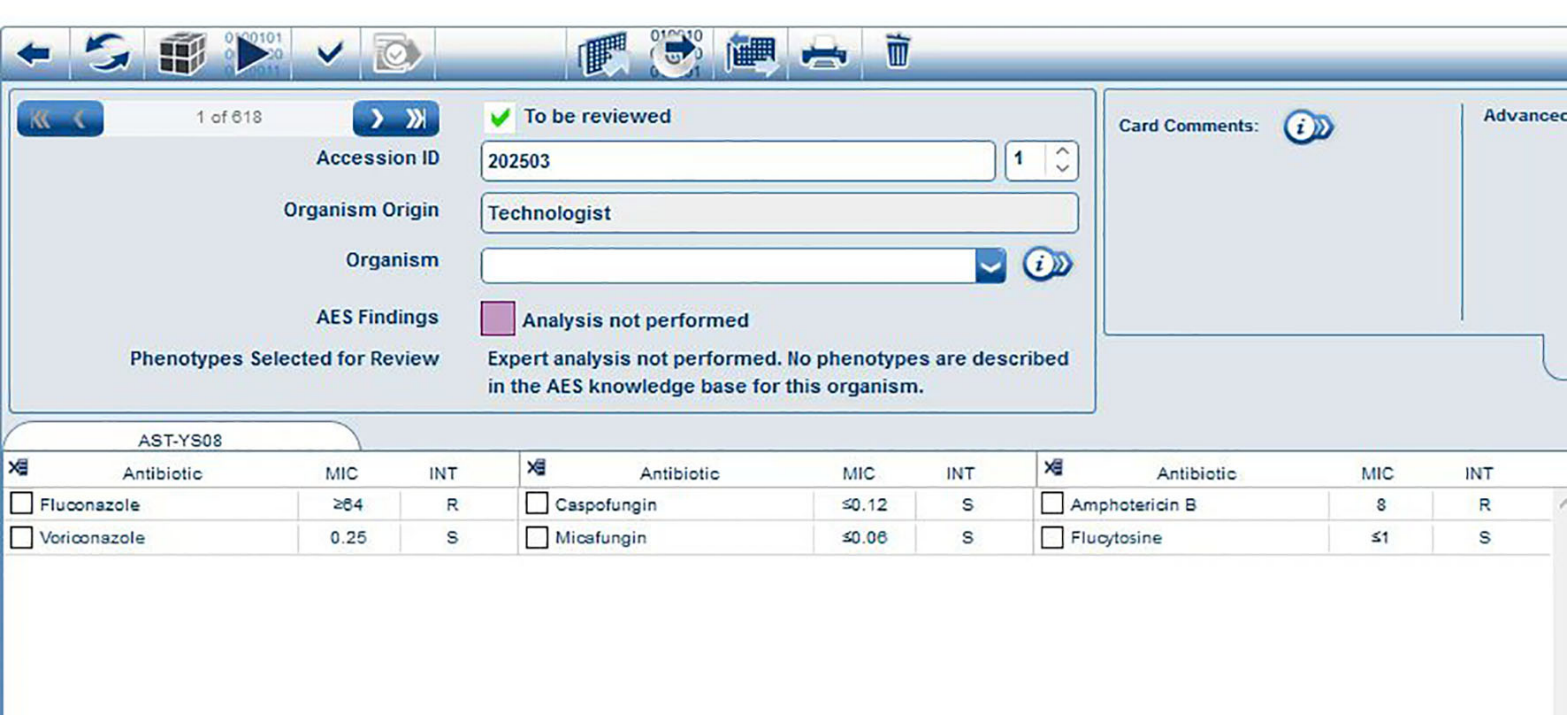
Antifungal Susceptibility Test Results of *C. auris* strain CAS20503.

The fungal susceptibility test results demonstrated that the strain was resistant to fluconazole (MIC ≥ 64 μg/ml) and amphotericin B (MIC = 8.0 μg/ml), while being sensitive to voriconazole (MIC = 0.25 μg/ml), caspofungin (MIC ≤ 0.12 μg/ml), micafungin (MIC ≤ 0.06 μg/ml), and flucytosine (MIC ≤ 1 μg/ml) (**Table 1**). The results of the MIC determined automatically by the VITEK 2 COMPACT system were shown in **Figure 4**.

### 3.4 Resistance gene profiling

For *ERG11*, 14 genes showed complete matches with the reference gene’s nucleic acid sequence, while 3 exhibited insertion or deletion variations. For *FKS1*, 3 genes perfectly matched the reference sequence, 4 had insertion or deletion variations, and 3 contained point mutations. For *ERG3*, 13 genes perfectly matched the reference sequence, 1 showed insertion or deletion variations, and 4 had point mutations. For *CDR1*, 4 genes perfectly matched the reference sequence, with 2 containing point mutations. For *MDR1*, 11 genes perfectly matched the reference sequence, 3 exhibited insertion or deletion variations, and 4 contained point mutations (**Table 2**).

**Table 2:** Table of drug-resistant gene distribution in *C. auris* strain CAS20503.

Antifungal Agents	Drug Resistance Gene	Gene ID	Number of Undetected Mutated Genes	Number of Genes with Insertions or Deletions (Indels)	Number of Genes with Point Mutations /(Gene Sequence Accession Number,Mutation site)
Triazoles (Fluconazole, Itraconazole, Voriconazole)	*ERG11*	40026303	14	3	0
Echinocandins (Cabozantine Mikafenjing)	*FKS1*	40026130	3	4	3/(ctg00076/89, Valine→Leucine), (ctg00078/478, Histidine→Proline), (ctg00080/85, Arginine→Serine)
Polyene (Amphotericin B)	*ERG3*	40028956	13	1	4/(ctg00020/218029, Leucine→Phenylalanine), ctg00027/137609, Leucine→ Phenylalanine), (ctg00045/68691, Leucine→Valine), (ctg00077/896, Asparticacid→Arginine)
Efflux Pump Gene	*CDR1*	40025314	4	0	2/(ctg00078/729, Phenylalanine→Cystine), (ctg00079/25, Tyrosine→Phenylalanine)
	*MDR1*	40029187	11	3	4/(ctg00007/210996, Lysine→Glutarnine; ctg00007/212121, Isoleucine→Valine), (ctg00024/72652, Phe Phenylalanine→Tyrosine), (ctg00075/16, Alanine→Proline), (ctg00082/15, Alanine→Proline)

### 3.5 Virulence gene prediction

Through gene prediction and alignment analysis, this study identified 10 virulence genes that met the screening criteria (E-value = 0 and query coverage > 60%). Details of their encoded proteins and functions are as follows:

Genes related to pathogenicity: *CaNik1* encoded histidine kinase, whose function was to promote hyphal formation and enhance virulence. *CHS2* encoded chitin synthase, which could promote hyphal formation and enhance virulence. *DUR1,2* encoded ureidoacidase, enabling *Candida* to enhance its colonization and survival abilities. *HSP90* encoded heat shock protein 90, which could promote the expression of hypha-related genes (e.g., *HWP1* and *ECE1*), contribute to biofilm formation, and assist *Candida* in immune evasion. *ICL1* encoded isocitrate lyase, which was a key determinant of virulence and associated with hyphal development. *PMT1* and *PMT2* encoded O-mannosyltransferase, which could maintain the integrity of the *Candida* cell wall, promote the formation of hyphae and biofilms, and enhance immune evasion ability. *PMT4* encoded O-mannosyltransferase, which could promote the glycosylation of virulence-related proteins and enhance the strain’s survival, invasion, and pathogenic abilities in the host. *SSD1* encoded an RNA-binding protein, which could maintain the integrity of the *Candida* cell wall and regulate *Candida*’s resistance to host antimicrobial peptides. *TPS2* encoded trehalose-6-phosphate phosphatase, which could promote hyphal formation and enhance virulence. The E-values, query coverage, and UniProt IDs of the above gene alignments were shown in **Table 3**.

**Table 3:** Virulence gene annotation of *C. auris* strain CAS20503 based on the DFVF database.

Gene Symbol	coding protein	E-value	query coverage (%)	UniProtID	function
*CaNik1*	Histidine Kinase	0	93	O42696_CANAL	Promote hyphal formation and enhance virulence.
*CHS2*	Chitin Synthase	0	98.6	Q5A409_CANAL	Promote hyphal formation and enhance virulence.
*DUR1,2*	Ureidoacidase	0	99.8	Q59VF3_CANAL	Enhance Candida colonization and survival.
*HSP90*	Heat Shock Protein 90	0	99.5	HSP90_CANAL	Promote the expression of hypha-associated genes (such as HWP1 and ECE1), facilitate biofilm formation, and assist Candida in escaping.
*ICL1*	Isocitrate Lyase	0	61.4	Q59RB8_CANAL	Key determinants of virulence and their association with hyphal development.
*PMT1*	O-mannosyltransferase	0	90	PMT1_CANAL	Maintain the integrity of the Candida cell wall, promote hyphal and biofilm formation, and enhance immune evasion.
*PMT2*	O-mannosyltransferase	0	94.8	Q5ADM9_CANAL	Maintain the integrity of the Candida cell wall, promote hyph
*PMT4*	O-mannosyltransferase	0	100	Q59X23_CANAL	Promote the glycosylation of virulence-related proteins, enhancing the strain's survival, invasion, and pathogenic capabilities within the host.
*SSD1*	RNA-binding protein	0	98.7	Q5AK62_CANAL	Maintain the integrity of the Candida cell wall and regulate Candida's resistance to host antimicrobial peptides.
*TPS2*	trehalose - 6 - phosphate phosphatase	0	98.3	Q5AI14_CANAL	Promote hyphal formation and enhance virulence.

### 3.6 Genes related to pathogenicity annotation

Based on the PHI-base database, a total of 6 pathogen-related genes were identified through screening with the criteria of E-value = 0 and query coverage > 95%. Among them, *CaCHS1* encoded chitin synthase, *ADE2* encoded phosphoribosylaminoimidazole carboxylase, *FAS2* encoded fatty acid synthetase, *PMR1* encoded a P-type ATPase transporter, *CaTPS2* encoded trehalose-6-phosphate phosphatase, and *Tfp1* encoded a putative subunit of V-ATPase. The coverage, PHI gene, and Protein ID corresponding to each gene were shown in **Table 4**.

**Table 4:** Virulence gene annotation of *C. auris* strain CAS20503 based on the PHI database.

GeneID	Protein ID	E-value	query coverage (%)	PHI_gene ID	Pathogen Gene	Gene Function
ctg00007_0001899_t	Q9URM1	0	100	CAA21947	*CaCHS1*	Chitin Synthase
ctg00007_0002011_t	Q92210	0	100	AAC49755	*ADE2*	Phosphoribosylaminoimidazole carboxylase
ctg00008_0002268_t	P43098	0	100	AAA34345	*FAS2*	Fatty acid synthetase
ctg00003_0000951_t	Q9P872	0	98.7	CAB87245	*PMR1*	P-type ATPase transporter
ctg00003_0000893_t	G1UAE0	0	98.3	CAC17748	*CaTPS2*	Trehalose-6-phosphate phosphatase
ctg00001_0000337_t	Q5AJB1	0	95.1	EAL02842	*Tfp1*	putative subunit of V-ATPase

### 3.7 Metabolic pathway annotation

**Figure 5:**
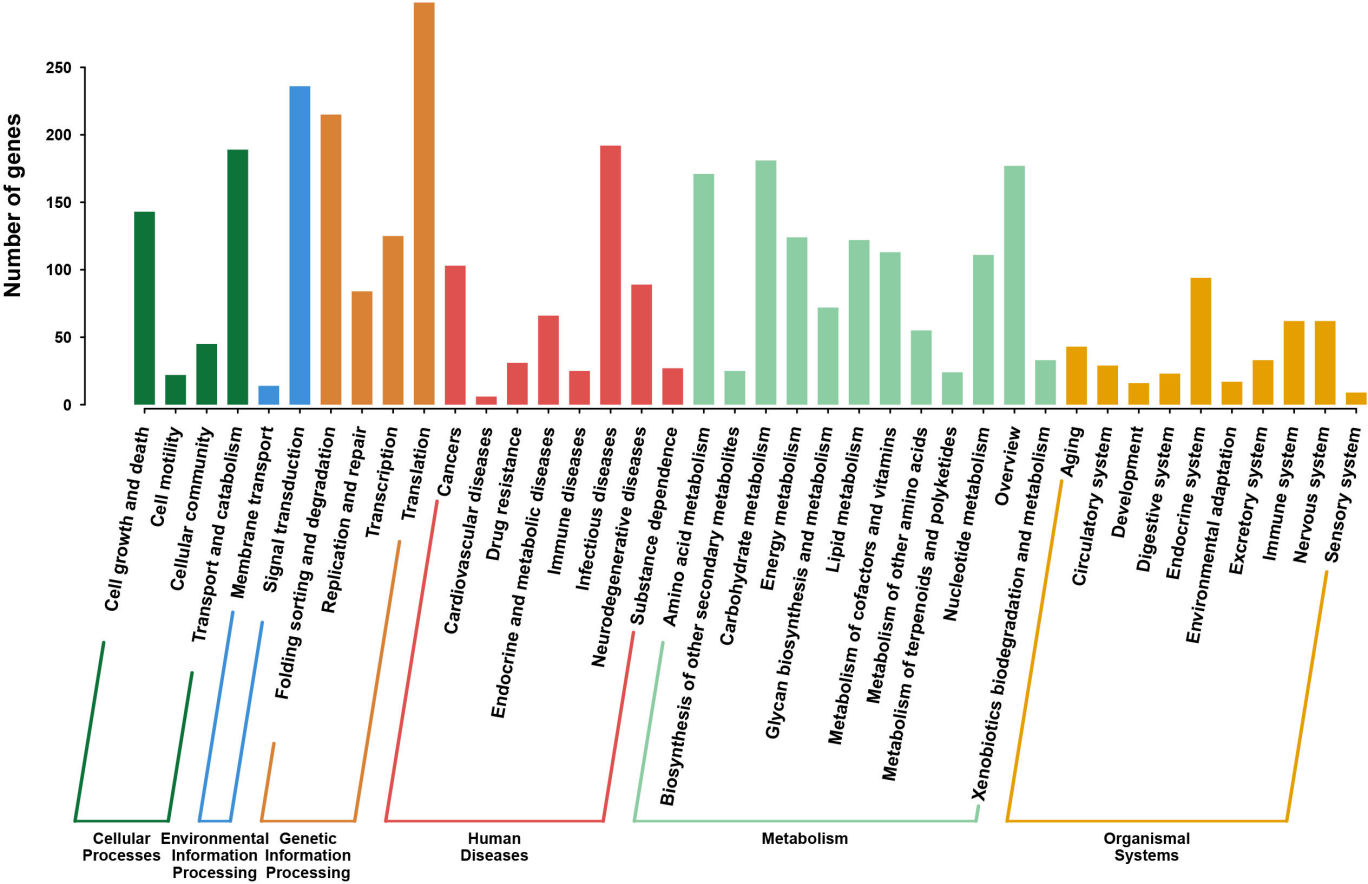
KEGG Pathway Classification of *C. auris* strain CAS20503. The pathway was composed of six branches. The vertical axis denotes the number of genes.The horizontal axis was categorized into six major functional branches in humans. Green: Cellular Processes, Dark Blue: Environmental Information Processing, Orange: Genetic Information Processing, Red: Human Diseases, Light Blue: Metabolism, Yellow: Organic Systems.

The pathways of the CAS20503 isolate annotated by the KEGG database consisted of 6 branches, namely: Cellular Processes, Environmental Information Processing, Genetic Information Processing, Human Diseases, Metabolism, and Organic Systems. KEGG pathway analysis indicated that among the annotated human disease categories, genes associated with infectious disease pathways were the most prominently expressed (**Figure 5**).

## 4 Phylogenetic analysis

**Figure 6:**
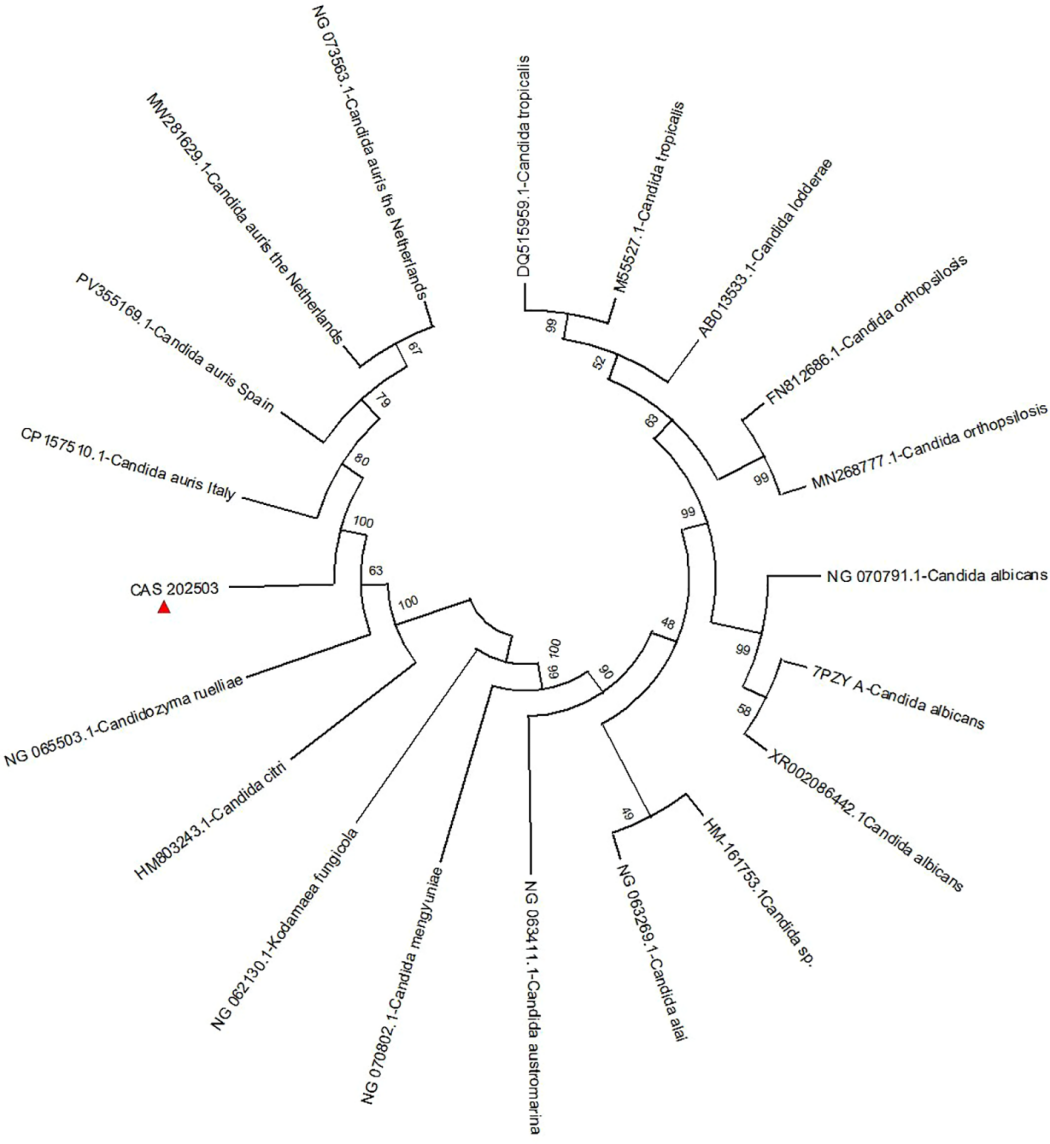
A phylogenetic tree was constructed through analysis of the 18S rRNA nucleotide sequence. The naming convention for strains in the figure follows the format: Assembly Accession ID + fungal name.The naming convention for *C. auris* strains includes the country of origin appended to the strain designation. The isolate CAS20503 is marked with a red triangle. Bootstrap values indicate branch support..

Phylogenetic reconstruction based on 18S rRNA homologous sequences from the core genome revealed that the strain clustered closely with other *C. auris* strains. Notably, it showed the highest sequence similarity to the *C. auris* strain belonging to clade I (a subset of the South Asian clade) isolated in Italy in 2024 (GenBank accession number: CP157510.1) (**Figure 6**).

## 5 Discussion

An analysis was conducted on the high-risk factors for *C. auris* infection in the patients involved in this study. *Candida* species were one of the main pathogens associated with fungal infections, particularly susceptible to infecting immunocompromised patients, as well as those who had been hospitalized for a long time or undergone invasive medical procedures (Halioti et al., 2024). Although *C. auris* was an emerging *Candida* species, the risk factors for patients infected with it were similar to those for infections caused by other Candida species (Biagi et al., 2019; Bradley, 2019). These factors included: advanced age; presence of indwelling medical devices (such as central venous catheters or urinary catheters); specific comorbidities (including diabetes mellitus, neoplastic diseases, and chronic kidney disease); receipt of total parenteral nutrition; dependence on mechanical ventilation; and receipt of hemodialysis (Biran et al., 2023). In addition, it had been documented that invasive infections could occur within 48 hours after patients were admitted to the intensive care unit (ICU) (Jeffery-Smith et al., 2017). In this study, the CAS20503 strain was isolated from the urine of an elderly male patient with multiple underlying diseases, including diabetes mellitus, hypertension, and renal insufficiency. The patient was admitted to the hospital due to knee joint effusion, and later transferred to the ICU due to acute respiratory failure. Five days later, *C. auris* was detected after urine culture was performed due to symptoms of fever and a significant decrease in urine output. The patient had been receiving long-term hemodialysis treatment due to renal insufficiency. Additionally, after being transferred to the ICU, he had a history of invasive procedures such as central venous catheterization, cystocentesis, endotracheal intubation, and ventilator support. These factors were likely the causes of the fungal infection in this patient (Rossow et al., 2021). Our findings highlight that clinicians should enhance fungal culture and surveillance for infected patients with underlying diseases, undergoing invasive treatment, and exhibiting poor response to antifungal agents, so as to avoid missed diagnosis.

In terms of identification, after 48 hours of culture on Sabouraud dextrose agar medium, the CAS20503 isolate formed smooth, milky white colonies. On CHROMagar Candida chromogenic medium, it formed pink, smooth, and moist colonies, which was consistent with literature reports (Cortegiani et al., 2018). However, these morphological characteristics of *C. auris* were similar to those exhibited by other common *Candida* species, so *in vitro* morphological evaluation of colonies alone was insufficient for accurate laboratory identification. Studies have reported that the VITEK 2 COMPACT system may produce false-negative results due to missed detection (Ambaraghassi et al., 2019). Since traditional morphological observation and biochemical identification methods often struggled to accurately identify *C. auris*, we were able to accurately and definitively identify *C. auris* through MALDI-TOF MS combined with genome sequencing technology. Therefore, for difficult or rare bacteria, especially in cases where traditional methods could not distinguish them from closely related *Candida* species, molecular detection technology was recommended (Satoh et al., 2009).

In the fungal drug susceptibility testing, the *C. auris* strain CAS20503 exhibited resistance to fluconazole and amphotericin B, and susceptibility to voriconazole, caspofungin, micafungin, and flucytosine. This was consistent with the known multidrug-resistant profile of *C. auris*. In the alignment of whole-genome nucleic acid sequences, key antifungal resistance genes were identified, including *ERG11*, *FKS1*, *ERG3*, *CDR1*, and *MDR1*. Among them, *ERG11* was associated with resistance to triazole drugs; *FKS1* was related to resistance to echinocandin drugs; *ERG3* was linked to resistance to polyene antibiotics; and *CDR1* and *MDR1*, which belonged to efflux pump genes, were involved in the active pumping of intracellular substances such as antibiotics out of the cell by pathogens, thereby leading to drug resistance. In addition, these genes harbored insertions, deletions, and point mutations, which might collectively contribute to the development of drug-resistant phenotypes. For instance, in the alignment of the *ERG11* gene, 3 insertion or deletion variations were identified. Such mutations altered the structure of lanosterol 14α-demethylase, reduced the binding capacity of triazole drugs, and thereby conferred fluconazole resistance in the strain (Jangir et al., 2023). The research results revealed that the *ERG3* gene contained 1 insertion or deletion variation and 4 point mutations; these mutations might affect ergosterol biosynthesis and impair the efficacy of amphotericin B by altering cell membrane integrity (Rybak et al., 2022; Carolus et al., 2024). The *FKS1* gene carried 4 insertion or deletion variations and 3 point mutations; these variations and mutations might disrupt the active site of 1,3-β-D-glucan synthase, which was further associated with decreased sensitivity to echinocandin drugs (Frras-De-LeLe et al., 2020; Lee et al., 2021). Moreover, mutations in the efflux pump genes *CDR1* (with 2 point mutations) and *MDR1* (with 3 insertion or deletion variations and 4 point mutations) might further enhance antifungal resistance (Li et al., 2024a; Li et al., 2024b).

Pathogenic fungi could cause a spectrum of diseases, ranging from superficial cutaneous and mucosal infections to deep-seated tissue and organ infections. In severe instances, such infections could result in multiple organ failure and even death (Denning, 2024). Through analysis of the virulence genes of the CAS20503 strain using the DFVF database, virulence-associated genes were identified. These genes might have exerted a synergistic effect during human infection by regulating key processes of C. auris, including morphological transformation, host colonization, immune evasion, and environmental adaptation, thereby significantly augmenting its pathogenicity and the persistence of infection. Among them, *CaNik1*, *CHS2*, *TPS2*, and *ICL1* synergistically promoted hyphal elongation, helping *Candida* penetrate host epithelial tissues and spread to the urinary system. *DUR1,2* combined with *PMT4* could jointly promote the colonization of *Candida* on the surface of the urethral mucosa. *HSP90* regulated the expression of biofilm-related genes to promote the synthesis of biofilm matrix and maintain the structural integrity of biofilms. It could synergize with *PMT1* and *PMT2* to enhance the ability of *Candida* to form biofilms on the surface of medical devices (such as urinary catheters and central venous catheters).

Based on the PHI-base database, a total of 6 pathogenicity-related genes were identified through the screening criteria of E-value = 0 and query coverage > 95%. They were *CaCHS1*, *ADE2*, *FAS2*, *PMR1*, *CaTPS2*, and *Tfp1*. During the process of *Candida* infecting the host, these 6 genes synergistically participated in the structural maintenance, substance metabolism, environmental adaptation, and virulence regulation of *Candida*. They enhanced its abilities of host colonization, tissue invasion, immune evasion, and persistent infection from multiple dimensions such as structural stability, metabolic support, and stress adaptation, and were important molecular bases for the pathogenic mechanism. KEGG pathway analysis showed that genes related to the metabolism of infectious diseases were enriched, indicating that the strain might have enhanced its own survival ability through specific metabolic adaptations during the process of host infection.

The drug resistance genes, virulence genes, and pathogenic genes of *C. auris* might have acted synergistically through a complex regulatory network to enhance its infectivity. Their drug resistance genes could help the strain resist clinical therapeutic interventions, virulence genes could improve its environmental adaptability and immune evasion ability, and pathogenic genes could provide guarantees for its survival and proliferation in the host. However, existing studies remained limited to the level of gene identification and functional speculation. Therefore, it was necessary to carry out *C. auris*-specific gene knockout or overexpression experiments to verify the actual functions of drug resistance genes and virulence genes.

In the treatment of *candidal* urinary tract infections, fluconazole was the first-choice drug (Escande4 et al., 2019). However, the resistance rate of *C. auris* to fluconazole was as high as 90%, and to voriconazole was 73% (Rudramurthy et al., 2017), making triazole drugs unsuitable for empirical treatment. Although the resistance rate of *C. auris* to echinocandins was lower than that to triazoles, resistance remained relatively common (Chowdhary et al., 2018). Literature reported that the resistance rate of *C. auris* isolates to amphotericin B was as high as 35% (Park et al., 2013). Given that *C. auris* strains might develop resistance to triazoles, echinocandins, and polyenes, it was necessary to consider alternative drugs in the treatment of urinary tract infections caused by *C. auris*. flucytosine was an attractive alternative. Compared with commonly used antifungal drugs, it was mainly excreted into urine in its active form, and according to existing studies, primary resistance of *C. auris* to flucytosine was uncommon (Charlier et al., 2015). The use of flucytosine in multidrug-resistant *C. auris* strains was consistent with current treatment guidelines for *Candida glabrata* (Pappas et al., 2016). The *C. auris* CAS20503 isolate in this study was susceptible to flucytosine, and flucytosine could be used for the treatment of urinary tract infection in this patient. In addition, combination therapy regimens had been used in multiple case reports for the treatment of *C. auris*, including echinocandins combined with amphotericin B, triazoles combined with echinocandins, or flucytosine combined with amphotericin B (Biagi et al., 2019; Bidaud et al., 2019; Ostrowsky et al., 2020). In the drug susceptibility testing of the CAS20503 isolate, the strain showed resistance to the triazole drug fluconazole and the polyene drug amphotericin B, which was consistent with the literature reports. Although the literature reported that the resistance rate of *C. auris* to voriconazole reached 73% (Rudramurthy et al., 2017), the isolate in this study was susceptible to voriconazole. Although the literature reported that resistance of *C. auris* to echinocandins was relatively common (Chowdhary et al., 2018), the CAS20503 isolate was susceptible to this class of antifungal drugs. Based on the results of fungal drug susceptibility testing and the treatment guidelines in the literature, clinicians selected caspofungin combined with flucytosine for treatment. Three weeks after treatment, the fungal culture turned negative, which demonstrated the clinical application value of individualized antifungal susceptibility testing. It had been pointed out in the literature that after the discontinuation of antifungal treatment, weekly cultures should be continued to guide the termination of infection control measures. Two negative culture results at one-week intervals were sufficient as the basis for terminating infection control measures (Griffith and Danziger, 2020). For the treatment of the CAS20503 isolate, clinical antifungal treatment and fungal monitoring were stopped after the fungal culture turned negative detected at three weeks of antifungal treatment. Therefore, there might be deficiencies in the continuous monitoring of fungi in clinical departments.

The genetic relatedness of *C. auris* strains could be assessed by multiple methods, including multi-locus sequence typing (MLST), proteomic analysis based on MALDI-TOF MS, and WGS (Girard et al., 2016). WGS remained the most advanced method for determining the clonality of *C. auris* strains (Hata et al., 2020; Kappel et al., 2024). Phylogenetic analysis based on 18S rRNA sequences in this study showed that the CAS20503 strain was genetically close to the *C. auris* strain with GenBank accession number CP157510.1, which was isolated in Italy in 2024 and belonged to clade I (a subset of the South Asian clade). The Italian isolate was a bloodstream infection isolate. Its drug resistance phenotype was identical to that of the CAS20503 isolate in that it was resistant to fluconazole and amphotericin B, and sensitive to caspofungin and micafungin. The difference was that the Italian isolate was resistant to flucytosine, while the CAS20503 isolate was sensitive to this drug. Both Candida auris isolates had mutations in the resistance genes *ERG11*, *ERG4*, and *CDR1*. The Italian isolate had the *FCY1* gene mutation associated with flucytosine resistance (Ben Abid et al., 2023), whereas no such mutated gene was identified in the CAS20503 isolate through alignment. This might have been the main reason why the isolate was sensitive to flucytosine.

Given the high pathogenicity and transmissibility of *C. auris*, it was advisable to strengthen hospital infection control measures (Aldejohann et al., 2022). After the strain was isolated, as it was the first time such a highly transmissible fungus had been found in the hospital, it attracted strong attention from physicians in the hospital infection control department. They required that the patient be placed in a single room and that standard contact precautions be implemented. All medical staff in contact with the patient were required to strictly follow hand hygiene protocols. Hospital infection control doctors regularly performed swab tests on the patient’s axillae, inguinal regions, and previous infection sites. Various surfaces in the ward were appropriately disinfected, including bedside tables, bed rails, windowsills, blood glucose meters, blood pressure cuff sleeves, crash carts, and nursing carts. In addition, the patient’s previous roommates and medical staff who had long-term contact with the patient also underwent swab tests to prevent the spread of infection to other susceptible patients.

## 6 Conclusion

An analysis of the *C. auris* strain CAS20503 in this study showed that its infection was closely associated with high-risk factors such as advanced age, underlying diseases, and invasive procedures, and clinicians needed to strengthen monitoring of such populations. Traditional methods struggled to achieve accurate identification, requiring a combination of MALDI-TOF MS and whole-genome sequencing. The strain was resistant to fluconazole and amphotericin B, while susceptible to caspofungin and others. The drug resistance phenotype was related to mutations in genes such as *ERG11* and *FKS1*, and the combined therapy guided by individualized drug susceptibility testing showed significant efficacy. Virulence and pathogenic genes might have synergistically enhanced infectivity by regulating colonization and biofilm formation, but this speculation still required further verification. The strain was genetically closely related to an Italian strain belonging to the South Asian clade, and they showed differences in susceptibility to flucytosine due to variations in the *FCY1* gene mutation. Although infection control measures such as single-room isolation were implemented, there were deficiencies in continuous clinical monitoring after treatment. In summary, this study provided references for the diagnosis, treatment, research on drug resistance mechanisms, and prevention and control of *C. auris*, highlighting the importance of accurate identification, individualized treatment, and systematic monitoring.

## 7 Study limitations

This study was based solely on the analysis of a single strain with a limited sample size. Furthermore, the functional speculation about drug resistance, virulence, and pathogenic genes was mainly based on literature reports on studies of *Candida albicans*. For the homologous genes identified in *C. auris* that share sequence similarity with these genes, we indeed only confirmed their existence through sequence alignment, and had not yet experimentally verified whether their functions were consistent with those of the homologous genes in *Candida albicans*. In view of this, it will be necessary for us to expand the sample size in the future and list “functional verification of genes in C. auris” as an important future research direction, so as to clarify their specific roles in the pathogenic process of this strain.

## Data Availability

The datasets presented in this study can be found in online repositories. The names of the repository/repositories and accession number(s) can be found in the article/supplementary material.
